# Periodontitis as a Modulator of Malignant Transformation in Oral Potentially Malignant Disorders: A Narrative Review of Biological Mechanisms and Clinical Implications

**DOI:** 10.1007/s11912-026-01817-z

**Published:** 2026-08-21

**Authors:** Giorgia Pieretto, Martina Sansavini, Arthur Rodriguez Gonzalez Cortes, Christian Bacci

**Affiliations:** 1 Department of Neurosciences, University of Padua, Padua, Italy; 2 Department of Biomedical and Neuromotor Sciences (DIBINEM), Martina Sansavini, University of Bologna, Bologna, Italy; 3 Department of Dental Surgery, Faculty of Dental Surgery, University of Malta, MSD 2080 Msida, Malta

**Keywords:** Periodontitis, Oral potentially malignant disorders, Oral squamous cell carcinoma, Oral microbiome, Chronic inflammation, Carcinogenesis

## Abstract

**Purpose of Review:**

Oral potentially malignant disorders (OPMDs) comprise a heterogeneous group of lesions with a variable risk of progression to oral squamous cell carcinoma (OSCC). Although conventional risk assessment relies primarily on clinical and histopathological features, increasing evidence suggests that the local oral microenvironment may also influence malignant transformation. This narrative review examines the potential role of periodontitis as a modulator of OPMD progression, with particular emphasis on the underlying biological mechanisms and clinical implications. A targeted literature search was conducted in PubMed/MEDLINE, Scopus/EMBASE, and Web of Science for studies published between January 2000 and March 2026.

**Recent Findings:**

Available evidence suggests that periodontitis may promote a pro-tumorigenic oral microenvironment through the interconnected effects of chronic inflammation, microbial dysbiosis, and metabolic alterations. These processes may enhance epithelial proliferation, genomic instability, immune evasion, and field cancerization, potentially facilitating the progression of OPMDs to OSCC. Although direct longitudinal and interventional evidence remains limited, converging mechanistic, biological, and epidemiological findings support the hypothesis that periodontal disease may contribute to early oral carcinogenic processes.

**Summary:**

Periodontitis may represent an underrecognized component of oral cancer risk, particularly in patients with OPMDs. Incorporating periodontal assessment into their clinical management may support more comprehensive risk stratification. Although causality has not been established, controlling periodontal inflammation and dysbiosis may offer a potential preventive strategy. Further longitudinal and interventional studies are required to determine the clinical relevance of this association.

**Graphical Abstract:**

**Figure:**
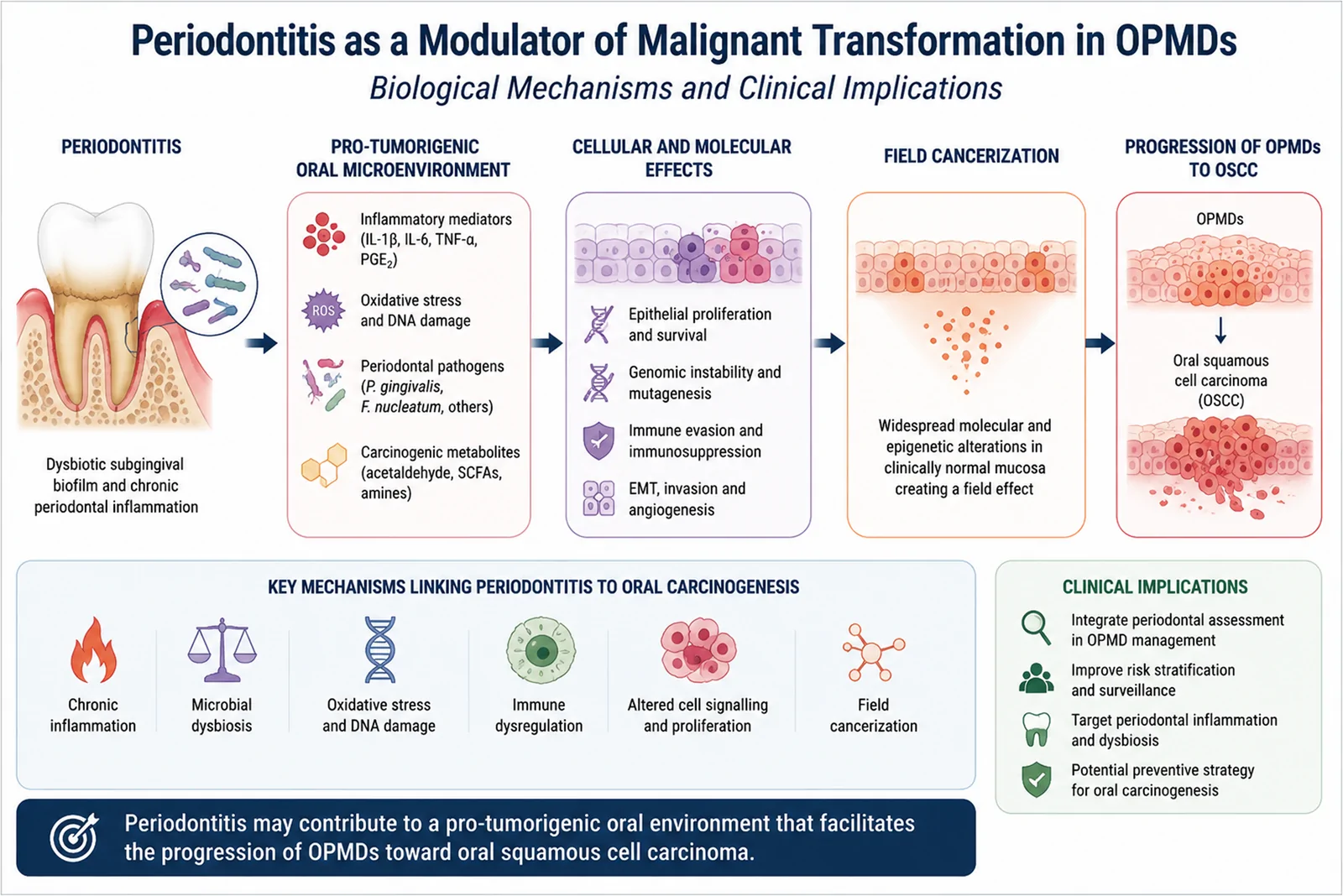

## Highlights

Periodontitis may contribute to a pro-tumorigenic oral microenvironment in OPMDs.

Chronic inflammation, oxidative stress, and microbial dysbiosis may influence malignant progression.

Periodontal pathogens can interact with epithelial signalling and immune regulatory pathways.

Field cancerization may be amplified by periodontal inflammation and microbiome alterations.

Periodontal assessment may support a more integrated approach to OPMD surveillance.

## Introduction

Oral potentially malignant disorders (OPMDs) refer to a heterogeneous group of lesions associated with an increased risk of malignant transformation into oral squamous cell carcinoma (OSCC), which remains a major contributor to global cancer morbidity and mortality [1, 2]. Despite advances in diagnostic and surveillance strategies, predicting malignant transformation at the individual level remains a significant challenge. Current risk assessment methods rely primarily on clinical and histopathological parameters, such as lesion subtype, anatomical location, and grade of epithelial dysplasia, along with exposure to established risk factors, including tobacco and alcohol use. However, these approaches do not fully capture the complex and dynamic biological processes underlying oral carcinogenesis [3–5].

In recent years, increasing scrutiny has been directed toward the role of the local mucosal field in cancer development. In this context, periodontitis, a chronic inflammatory disease driven by a dysbiotic oral microbiome, has emerged as a potential contributor to oral carcinogenic pathways. Recent epidemiological and experimental evidence supports an association between periodontal disease and OSCC, suggesting that persistent inflammation, immune dysregulation, and microbial activity may collectively contribute to tumor initiation and progression [6–11]. In particular, periodontal pathogens such as *Porphyromonas gingivalis* and *Fusobacterium nucleatum* have been shown to interfere with epithelial cell signaling, enhance proliferative capacity, inhibit apoptosis, and modulate the tumor microenvironment, thereby promoting oncogenic processes [12–16]. Nevertheless, although the link between periodontitis and established OSCC has been increasingly recognized, its potential role in earlier stages of oral carcinogenesis remains insufficiently investigated.

The transition from OPMDs to invasive carcinoma represents a biologically critical phase in which local environmental factors, including chronic inflammation [17, 18] and microbial dysbiosis [19–22], may influence malignant evolution. Emerging evidence suggests that the oral microbiome and inflammatory profile differ significantly among healthy mucosa, OPMDs, and OSCC, indicating a progressive ecological and molecular shift during malignant transformation [23]. Within this framework, the concept of field cancerization provides a useful basis for interpreting these processes [24, 25]. Beyond the accumulation of genetic alterations, field cancerization reflects the presence of widespread epithelial changes driven by both intrinsic and extrinsic factors. Increasing evidence suggests that inflammation and microbial dysbiosis may contribute to this field effect, raising the possibility that periodontitis may help shape a pro-tumorigenic oral environment [26–28].

**Fig. 1:**
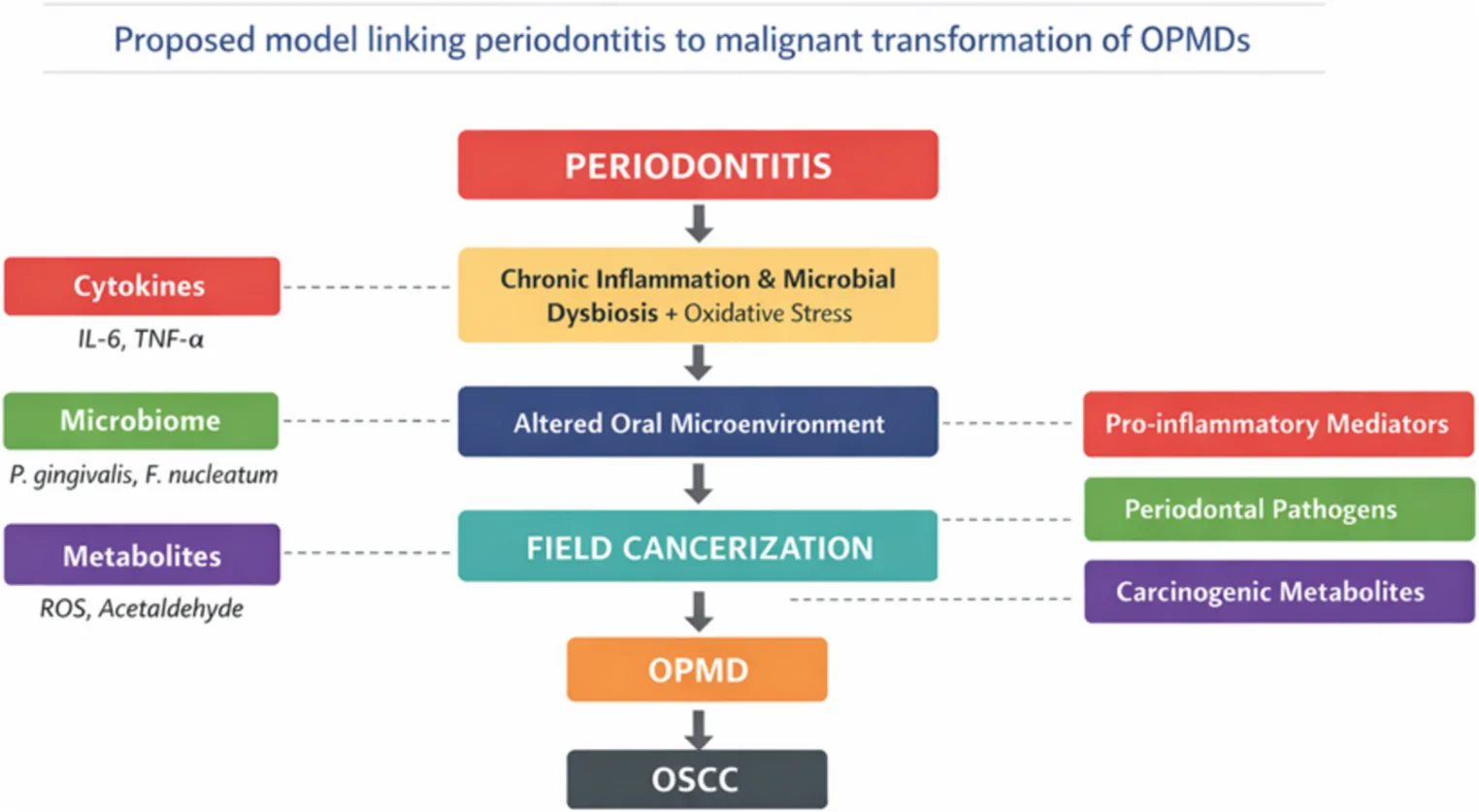
Proposed model linking periodontitis to malignant transformation of oral potentially malignant disorders (OPMDs). Periodontitis may contribute to a pro-tumorigenic oral microenvironment through the interplay of chronic inflammation, microbial dysbiosis, and oxidative stress. These processes are associated with the release of pro-inflammatory cytokines (e.g., IL-6, TNF-α), the activity of periodontal pathogens (e.g., Porphyromonas gingivalis, Fusobacterium nucleatum), and the production of carcinogenic metabolites (e.g., reactive oxygen species and acetaldehyde). Together, these factors may promote epithelial alterations and contribute to field cancerization, potentially facilitating the progression from OPMDs to oral squamous cell carcinoma (OSCC)

Against this background, this narrative review aimed at exploring the potential role of periodontitis in the malignant transformation of OPMDs. By integrating current evidence on chronic inflammation, microbial dysbiosis, and field cancerization, this review seeks to provide a conceptual framework for understanding how periodontal disease may influence early carcinogenic processes, with possible implications for risk stratification, prevention, and clinical management. Our hypothetical framework linking periodontitis to malignant transformation in OPMDs is illustrated in Fig. 1.

## Materials and methods

**Fig. 2:**
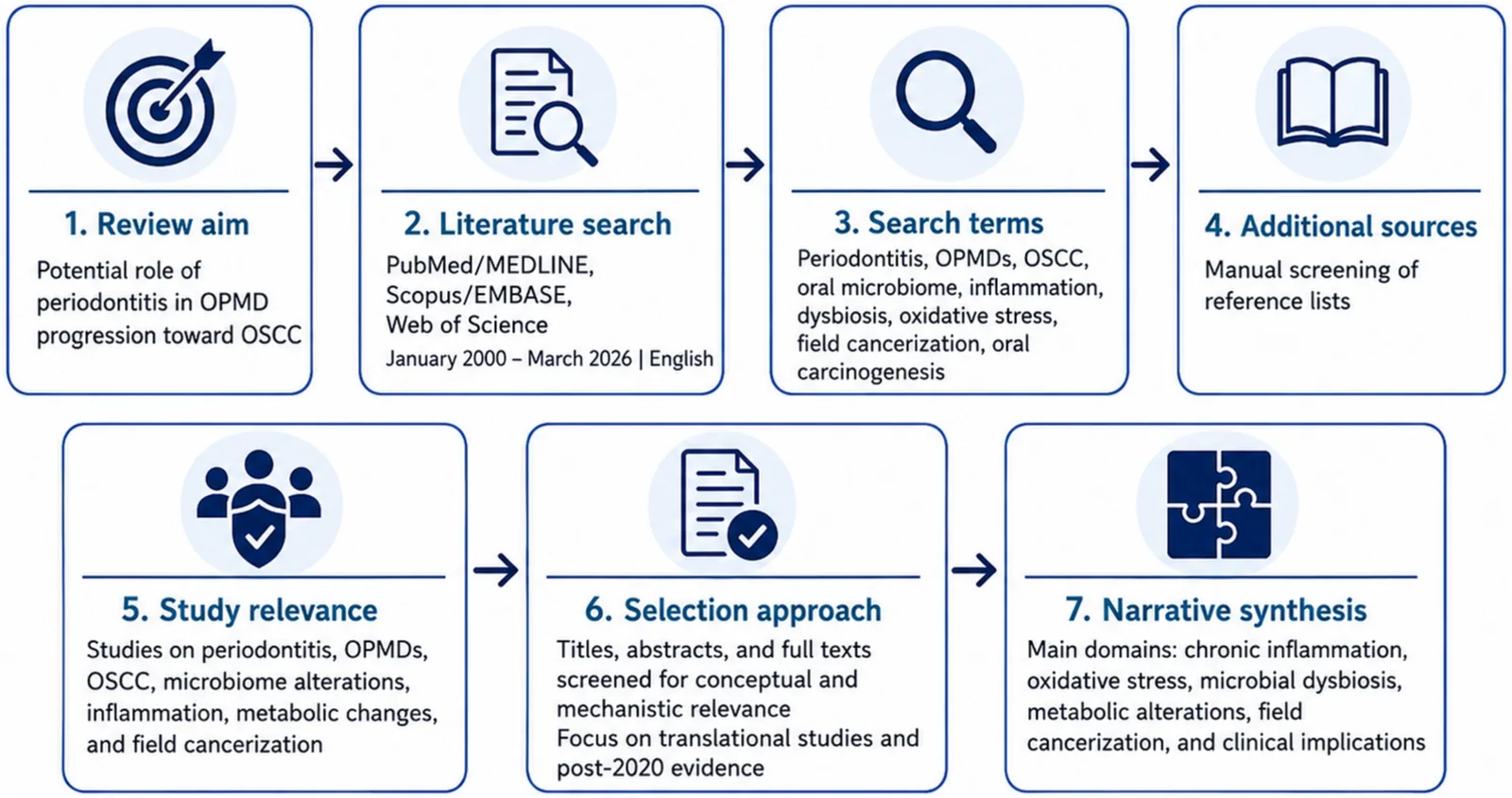
Overview of the targeted literature selection process used for this narrative review. Articles were selected according to conceptual and mechanistic relevance rather than formal systematic review criteria

This narrative review was conducted using a structured literature search to examine the potential contribution of periodontitis to the progression of oral potentially malignant disorders (OPMDs) to oral squamous cell carcinoma (OSCC). The review focused on biological and clinical mechanisms potentially linking periodontal inflammation, oral microbial dysbiosis, metabolic alterations, field cancerization, and malignant transformation. A targeted search of the literature was performed in PubMed/MEDLINE, Scopus/EMBASE, and Web of Science. The search included articles published in English between January 2000 and March 2026. The following keywords and their combinations were used: “*periodontitis*”, “*periodontal disease*”, “*oral potentially malignant disorders*”, “*oral epithelial dysplasia*”, “*oral leukoplakia*”, “*oral squamous cell carcinoma*”, “*oral microbiome*”, “*chronic inflammation*”, “*dysbiosis*”, “*oxidative stress*”, “*field cancerization*”, and “*oral carcinogenesis*”. The reference lists of relevant articles were also manually screened to identify additional studies of conceptual or mechanistic relevance. Eligible sources included original clinical studies, observational studies, experimental studies, translational studies, systematic reviews, meta-analyses, and authoritative narrative reviews. Studies were considered relevant when they addressed at least one of the following topics: the association between periodontitis and OSCC; the role of periodontal inflammation in carcinogenesis; oral microbiome alterations in OPMDs or OSCC; microbial or metabolic mechanisms potentially involved in malignant transformation; or the contribution of the local oral microenvironment to field cancerization. Seminal studies were also included when necessary to contextualise key concepts. Titles and abstracts were initially screened for relevance. Full texts were then assessed when the article appeared to contribute directly to the biological, clinical, or conceptual framework of the review. Particular attention was given to studies providing mechanistic or translational insights and to recent evidence published after 2020. Studies not directly related to periodontitis, OPMDs, OSCC, oral microbiome alterations, or oral carcinogenic mechanisms were not prioritised for inclusion. Because this was a narrative review, study selection was based on conceptual and mechanistic relevance rather than on predefined systematic review criteria. Therefore, no formal risk-of-bias assessment, quality scoring, meta-analysis, or quantitative synthesis was performed. However, to improve transparency and reproducibility, the search strategy, eligibility criteria, and thematic selection process were defined a priori. The available evidence was then narratively synthesised according to the main biological domains emerging from the literature: chronic inflammation, oxidative stress, microbial dysbiosis, metabolic alterations, field cancerization, and clinical implications for OPMD management. The study selection process is illustrated in Fig. 2.

## Biological Link Between Periodontitis and Carcinogenesis

Periodontitis is characterized by a chronic, dysregulated host response to a polymicrobial biofilm, resulting in sustained inflammation and progressive tissue destruction [29, 30]. Beyond its local effects, this persistent inflammatory state has increasingly been implicated in carcinogenesis through interconnected biological mechanisms involving immune modulation, oxidative stress, and microbial-host interactions [31]. In this context, periodontitis should not be interpreted as an isolated carcinogenic trigger, but rather as a chronic inflammatory condition capable of shaping a local biological environment that may favor early tumor-promoting processes.

Chronic inflammation represents a central driver of this association. Periodontal lesions are characterized by increased levels of pro-inflammatory mediators, including interleukin-6 (IL-6), tumor necrosis factor-alpha (TNF-α), and prostaglandins, which may promote epithelial proliferation, inhibit apoptosis, stimulate angiogenesis, and contribute to tissue remodelling [32, 33]. These processes overlap with well-recognized tumor-promoting inflammatory pathways, supporting the hypothesis that periodontal inflammation may contribute to a permissive microenvironment for oral carcinogenesis [34, 35]. Sustained inflammatory signaling may be particularly relevant in tissues where epithelial homeostasis is already altered, as occurs in oral potentially malignant disorders (OPMDs), where local biological stressors may influence lesion persistence and progression [27, 36–38].

Oxidative stress provides an additional mechanistic link. Chronic periodontal inflammation is associated with increased production of reactive oxygen and nitrogen species, which may induce DNA damage, genomic instability, and epigenetic alterations, all of which are involved in cancer development [39–41]. Repeated exposure of oral epithelial tissues to oxidative and inflammatory stress may therefore favor the accumulation of molecular alterations associated with malignant transformation. Rather than acting independently, these mechanisms may interact with pre-existing epithelial abnormalities and contribute to a tissue context in which early carcinogenic events are more likely to persist.

Host–microbe interactions represent a further component of this biological interface. Periodontitis is associated with a dysbiotic microbial community enriched in pathogenic species, including *Porphyromonas gingivalis* and *Fusobacterium nucleatum*, both of which have been implicated in cancer-related pathways [42–44]. These microorganisms and their virulence factors may influence epithelial and immune cell behaviour by modulating pathways involved in cell survival, proliferation, immune evasion, and tissue invasion [45, 46]. However, their role should be interpreted within a broader ecological and inflammatory context, rather than as the effect of individual pathogens alone. The specific contribution of microbial dysbiosis and metabolic alterations to oral carcinogenesis is discussed in greater detail in the following sections.

**Fig. 3:**
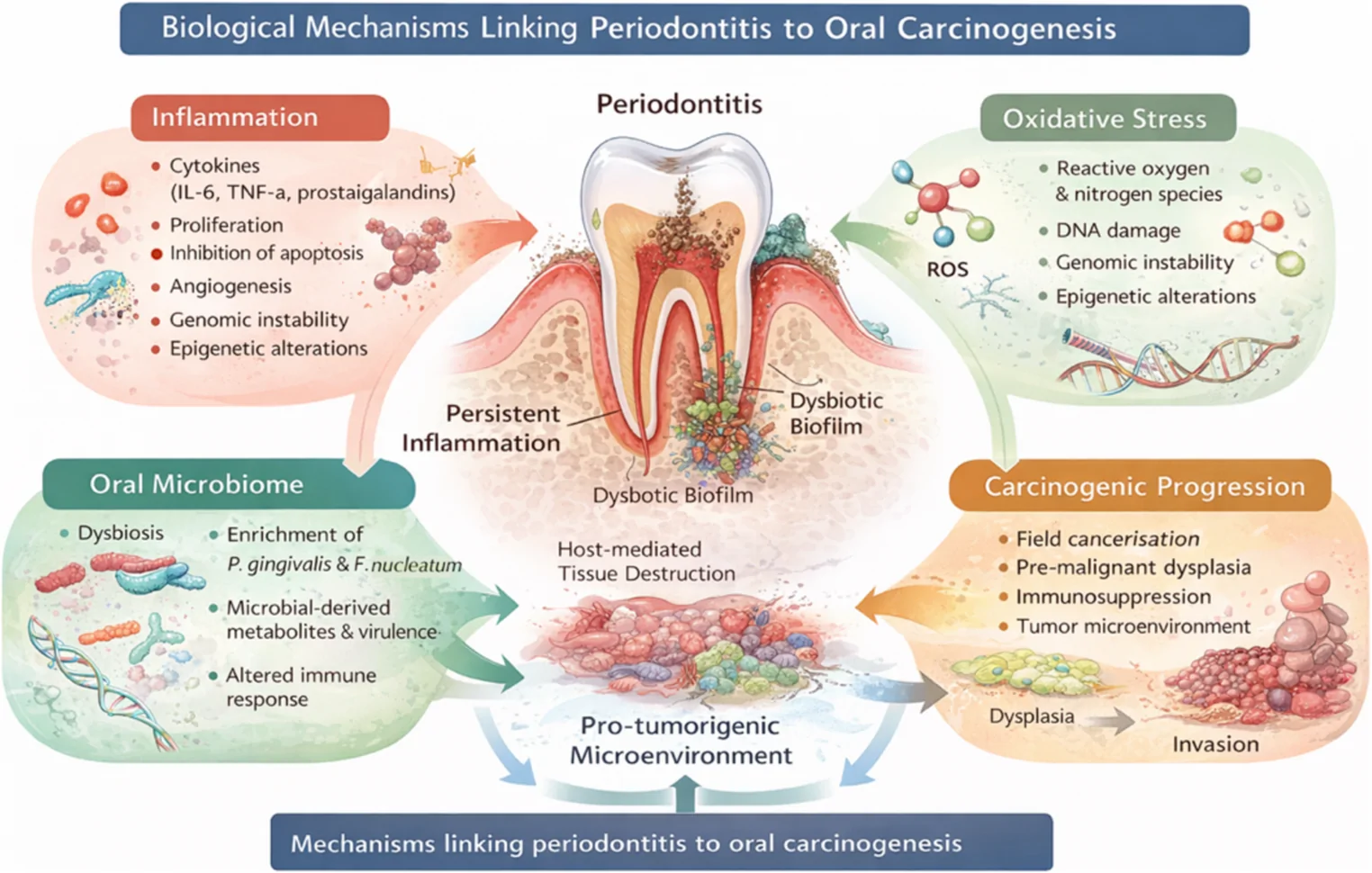
Biological mechanisms linking periodontitis to oral carcinogenesis. Chronic inflammation, oxidative stress, oral microbial dysbiosis, and immune dysregulation interact to create a pro-tumorigenic microenvironment. Through cytokine release, reactive oxygen species production, microbial virulence factors, and altered host responses, periodontitis may promote epithelial instability and facilitate carcinogenic progression

Overall, current biological and translational evidence suggests that periodontitis may act as a contextual amplifier of carcinogenic mechanisms by sustaining chronic inflammation, oxidative stress, epithelial instability, and dysregulated host–microbe interactions [47–49]. This model is particularly relevant to the transition from OPMDs to OSCC, where early microenvironmental alterations may influence the biological trajectory of already altered epithelial fields. The main biological pathways through which periodontitis may contribute to oral carcinogenesis are schematically illustrated in Fig. 3 and summarised in Table 1.

**Table 1:** Biological mechanisms linking periodontitis to oral carcinogenesis

Mechanism	Key mediators / factors	Biological effects	Potential impact on OPMDs progression
Chronic inflammation	IL-6, TNF-α, PGE2	Increased cell proliferation, inhibition of apoptosis, angiogenesis	Promotion of dysplastic progression
Oxidative stress	Reactive oxygen species (ROS), reactive nitrogen species	DNA damage, genomic instability, epigenetic alterations	Facilitation of malignant transformation
Microbial dysbiosis	*Porphyromonas gingivalis*, *Fusobacterium nucleatum*	Immune modulation, epithelial-mesenchymal transition (EMT), immune evasion	Enhancement of tumor-promoting pathways
Microbial metabolites	Short-chain fatty acids, acetaldehyde	Cellular toxicity, mutagenesis, altered signaling pathways	Increased carcinogenic potential
Immune dysregulation	Cytokine imbalance, altered host response	Chronic immune activation, impaired immune surveillance	Reduced control of abnormal epithelial cells
Field cancerization (indirect mechanism)	Combined inflammatory and microbial effects	Expansion of genetically altered epithelial fields	Increased risk of multifocal transformation

## Field Cancerization and the Oral Microenvironment

The concept of field cancerization provides a fundamental framework for understanding the spatial and temporal dynamics of oral carcinogenesis. Initially described as the presence of genetically altered epithelial fields extending beyond clinically detectable lesions, this concept has evolved to encompass a broader range of molecular and microenvironmental alterations that predispose to malignant transformation [24, 25, 50]. In the context of oral potentially malignant disorders (OPMDs), field cancerization helps explain both lesion heterogeneity and variability in clinical behaviour. While traditional models of field cancerization have mainly focused on cumulative exposure to carcinogens such as tobacco and alcohol, increasing attention has been directed towards the role of local biological factors, including inflammation and microbial dysbiosis, in shaping the carcinogenic field [51, 52]. In this regard, periodontitis represents a persistent local source of inflammatory mediators and pathogenic microorganisms within the oral cavity, potentially contributing to the establishment and expansion of altered epithelial fields [31, 53].

The periodontal microenvironment is characterized by sustained immune activation, increased vascular permeability, and continuous epithelial turnover, conditions that may facilitate the accumulation and propagation of genetic and epigenetic alterations in adjacent mucosal tissues. Pro-inflammatory cytokines and reactive oxygen species generated in periodontal lesions may diffuse beyond the immediate site of infection, contributing to a broader field of epithelial damage and genomic instability [38, 54]. These findings suggest that the biological impact of periodontitis may extend beyond periodontal tissues and influence the behaviour of nearby OPMDs. Microbial factors may further reinforce this field effect. Dysbiotic communities associated with periodontitis are not confined to periodontal pockets but may also colonise mucosal surfaces, thereby altering epithelial–microbial interactions across a wider area of the oral cavity [55, 56]. Pathogens such as *P. gingivalis* and *F. nucleatum* can modulate host cell signaling, disrupt epithelial barrier function, and promote immune evasion, contributing to a microenvironment conducive to malignant transformation [13, 14]. More recent evidence has also highlighted the role of microbiome-driven metabolic alterations in sustaining pro-tumorigenic conditions within the oral ecosystem [57, 58]. From this perspective, periodontitis may act as a co-driver in field cancerization by amplifying both inflammatory and microbial influences on epithelial tissues [26]. This view suggests that malignant transformation may result not only from intrinsic epithelial alterations, but also from dynamic interactions with the surrounding microenvironment.

**Fig. 4:**
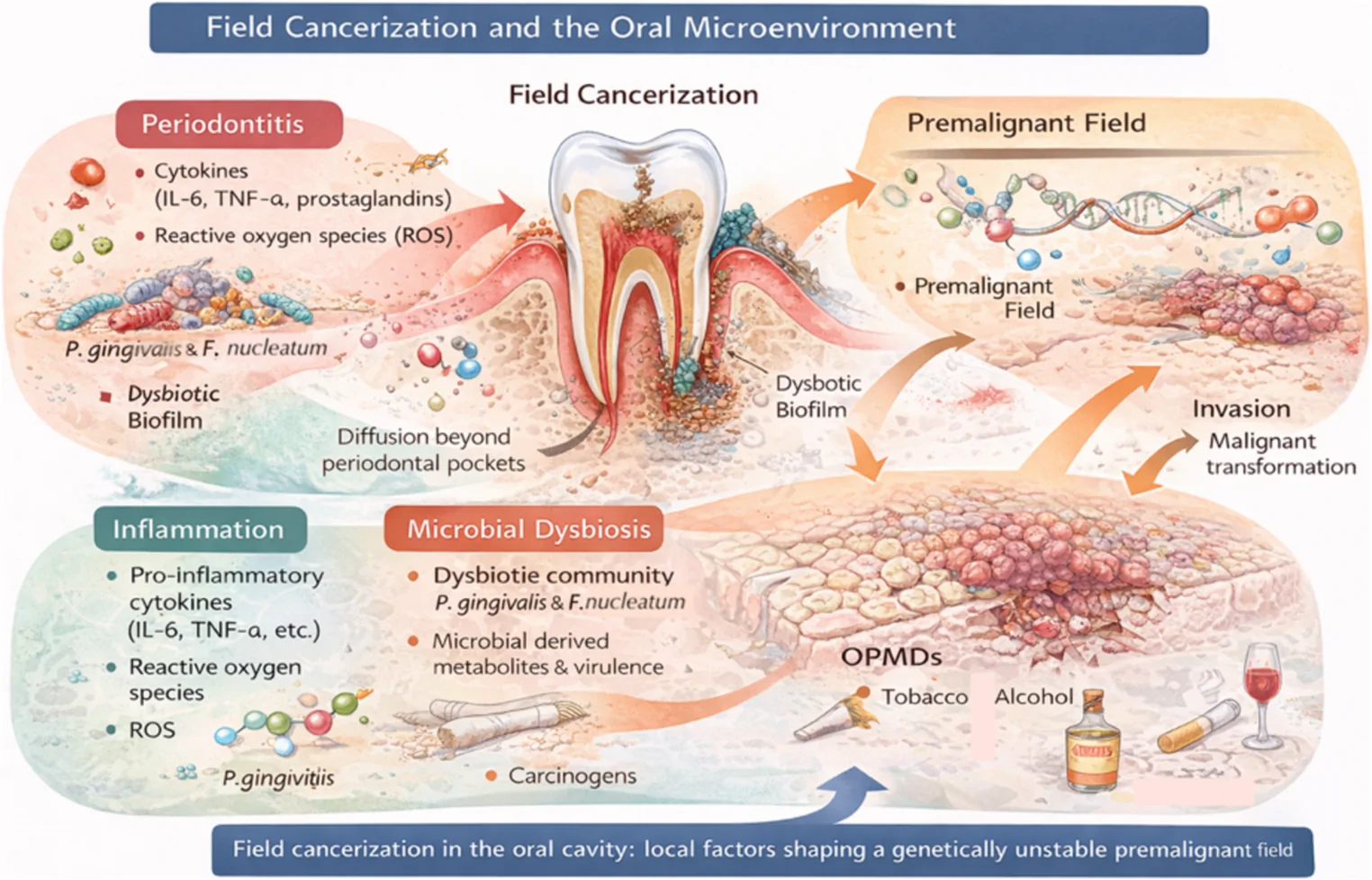
Schematic representation of the mechanism of field cancerization amplified by inflammatory processes associated with periodontal disease

Clinically, integrating periodontal status into the framework of field cancerization may help explain the variability in OPMD progression and may improve risk stratification. Lesions arising within a chronically inflamed and dysbiotic environment may exhibit a higher propensity for malignant transformation compared to those in more stable conditions. Although direct longitudinal evidence remains limited, this hypothesis is increasingly supported by converging biological and epidemiological data [48, 59]. The role of periodontitis in amplifying field cancerization and shaping a biologically altered oral microenvironment is schematically represented in Fig. 4.

## Microbiome and Metabolic Alterations in Oral Carcinogenesis

The oral microbiome is increasingly recognized as an active component of the oral carcinogenic process rather than a passive bystander. Through continuous interactions with epithelial cells, immune mediators, and local metabolites, the microbial ecosystem of the oral cavity may influence epithelial homeostasis, inflammatory responses, and cancer-related signaling pathways.

**Fig. 5:**
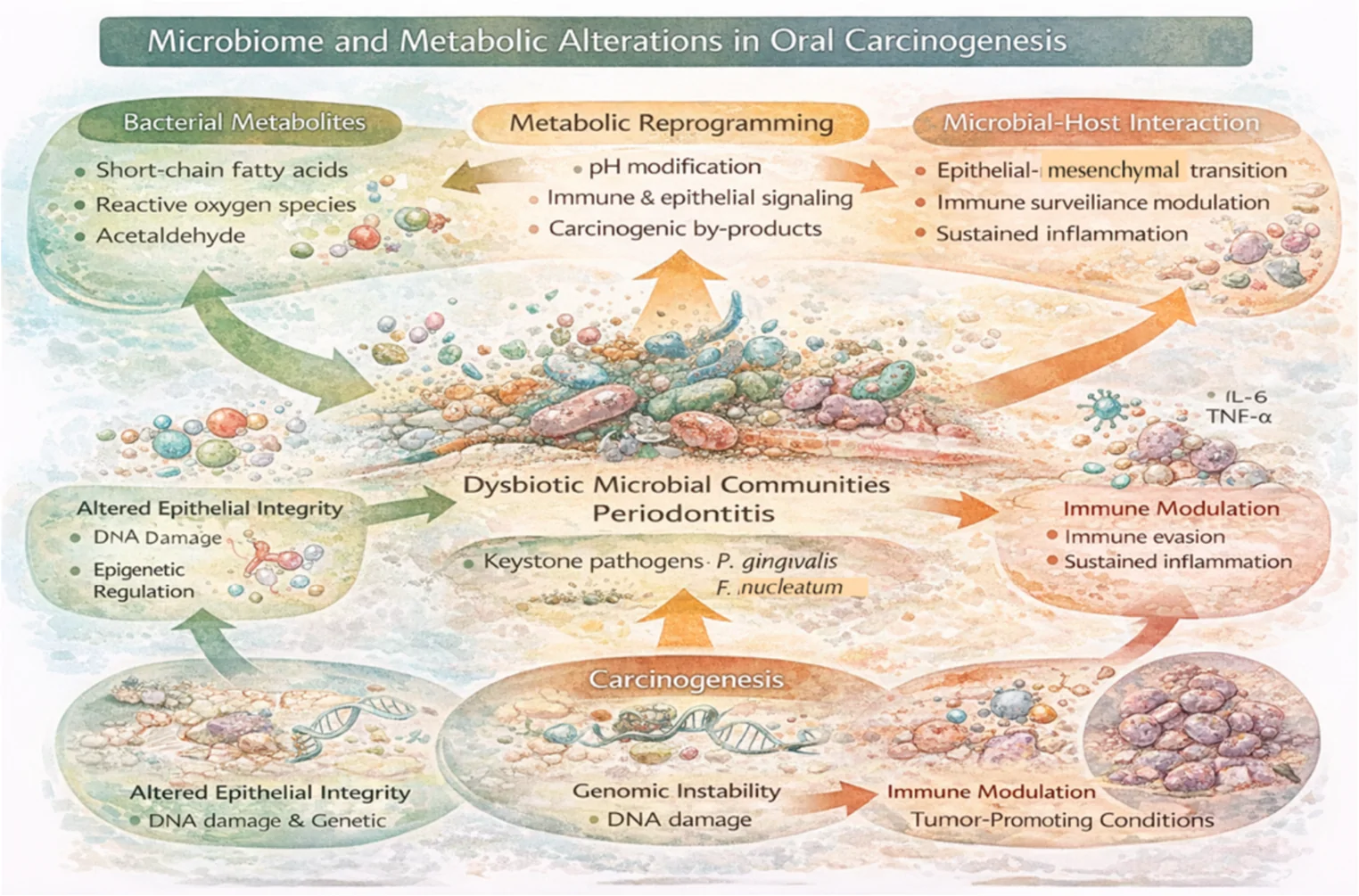
Microbiome and metabolic alterations in oral carcinogenesis. Periodontitis-associated dysbiosis may favor carcinogenesis through compositional and functional changes in the oral microbiome. Microbial virulence factors and metabolic by-products, including short-chain fatty acids, reactive oxygen species, and acetaldehyde, may alter epithelial homeostasis, immune responses, and genomic stability, thus contributing to a carcinogenic oral ecosystem

In this context, periodontitis-associated dysbiosis is of particular interest, as it represents a persistent ecological shift towards microbial communities with pro-inflammatory and potentially tumor-promoting properties [26–28, 46, 60]. The broader interplay between microbial dysbiosis, metabolic alterations, and tumor-promoting pathways is illustrated in Fig. 5.

Recent studies have shown that the oral microbiome differs across healthy mucosa, OPMDs, and OSCC, suggesting that microbial changes may accompany the transition from normal epithelial homeostasis to malignant disease [59, 61–63]. These differences involve not only microbial diversity and relative abundance, but also functional changes in microbial activity [62]. Such findings support the concept of a progressive ecological shift during oral carcinogenesis, although the directionality of this relationship remains uncertain. In particular, it is still unclear whether dysbiosis actively contributes to malignant progression, reflects epithelial and immune changes occurring during disease evolution, or represents a combination of both processes.

Within this dysbiotic ecosystem, keystone periodontal pathogens such as *Porphyromonas gingivalis* and *Fusobacterium nucleatum* have received particular attention because of their ability to interact with host epithelial and immune cells [42, 44, 64]. These microorganisms may modulate cancer-related pathways involved in epithelial–mesenchymal transition, immune evasion, cell survival, and tissue invasion [65, 66]. However, their effects should not be interpreted as isolated pathogen-specific mechanisms. Rather, they are likely to depend on the broader microbial community, host inflammatory status, epithelial susceptibility, and exposure to established carcinogenic factors. Beyond compositional changes, the metabolic activity of dysbiotic microbial communities may provide a functional link between periodontitis and carcinogenesis.

**Fig. 6:**
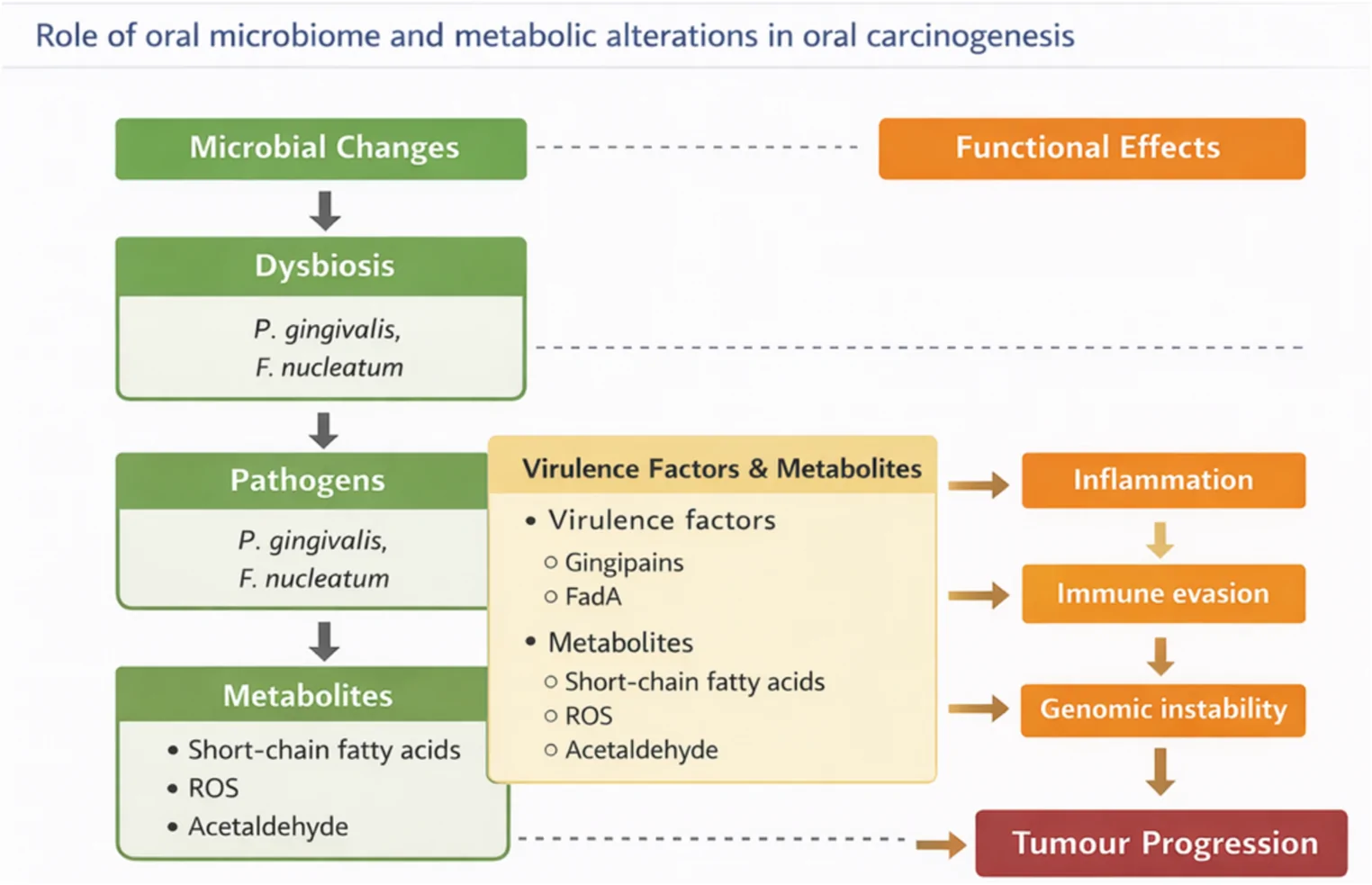
Role of oral microbiome and metabolic alterations in oral carcinogenesis. Dysbiosis of the oral microbiome, characterized by the enrichment of periodontal pathogens such as Porphyromonas gingivalis and Fusobacterium nucleatum, contributes to the production of virulence factors and carcinogenic metabolites, including short-chain fatty acids, reactive oxygen species (ROS), and acetaldehyde. These microbial-derived products can promote inflammation, immune evasion, and genomic instability, thereby facilitating tumor progression. This schematic representation highlights the functional link between microbial alterations and host cellular responses in oral carcinogenesis

Periodontitis-associated microbiota can generate bioactive products, including short-chain fatty acids, reactive oxygen species, and acetaldehyde, which may influence epithelial integrity, redox balance, immune responses, and genomic stability [67]. These microbial-derived products may be especially relevant in OPMDs, where epithelial homeostasis is already altered and additional inflammatory or metabolic stressors could contribute to lesion persistence or progression. These interactions are schematically illustrated in Fig. 6.

Microbial metabolism may therefore represent a bridge between dysbiosis, inflammation, and epithelial transformation. Bacterial-derived metabolites can modify local pH, affect oxidative balance, and influence epigenetic regulation in host cells, thereby contributing to a biologically permissive environment for tumor initiation and progression [68, 69]. More recent evidence further suggests that microbiome-driven metabolic reprogramming may interact with host pathways involved in proliferation, apoptosis, immune surveillance, and tissue remodelling [49, 70, 71]. This supports a bidirectional model in which microbial communities influence the host microenvironment while, at the same time, epithelial and inflammatory changes reshape the microbiome.

From a spatial perspective, periodontitis-associated dysbiosis may not remain confined to periodontal pockets. Dysbiotic communities and their metabolic products may affect adjacent mucosal surfaces and contribute to a broader ecological imbalance within the oral cavity [72, 73]. This observation is consistent with the concept of field cancerization, in which local inflammation, microbial exposure, and epithelial alterations may interact across a wider mucosal field. In this sense, the oral microbiome may be considered part of a dynamic carcinogenic network involving periodontal inflammation, epithelial susceptibility, environmental exposures, and host immune responses.

Despite these advances, the temporal and causal relationships between microbiome alterations and malignant transformation remain insufficiently defined. Most available studies are cross-sectional, limiting the ability to determine whether dysbiosis is a driver of progression, a consequence of epithelial alteration, or both. In addition, heterogeneity in sampling sites, sequencing platforms, analytical pipelines, periodontal definitions, and OPMD classification complicates comparisons across studies [74]. These limitations are particularly important because microbial signatures may vary according to lesion type, oral site, disease stage, smoking status, oral hygiene, and periodontal condition.

Nevertheless, integrating microbiome and metabolic profiling into the study of OPMDs represents a promising direction for future research. Identifying microbial signatures or metabolic patterns associated with high-risk lesions could improve biological risk stratification and generate testable hypotheses for preventive strategies. At present, however, targeting periodontal dysbiosis should be considered a biologically plausible but still unproven approach to modifying the oral microenvironment in patients with OPMDs. Robust longitudinal and interventional studies are required before microbiome-based strategies can be translated into clinical recommendations.

Taken together, current evidence suggests that microbiome and metabolic alterations may contribute to a biologically permissive oral environment and may eventually serve as biomarkers of disease progression. However, their role as direct drivers of malignant transformation in OPMDs remains to be demonstrated.

## Periodontitis in the OPMD → OSCC Transition

While the association between periodontitis and established oral squamous cell carcinoma (OSCC) has been increasingly documented, its role in the intermediate phase of carcinogenesis, namely the transition from oral potentially malignant disorders (OPMDs) to invasive carcinoma, remains largely underexplored. This gap is particularly relevant from a clinical perspective, as it concerns the stage at which preventive and therapeutic interventions may have the greatest impact.

Indeed, understanding which local factors may favor malignant progression before invasive disease becomes clinically evident could substantially improve surveillance strategies and open new opportunities for risk-adapted management. The progression from OPMDs to OSCC is widely recognized as a multifactorial process driven by cumulative genetic alterations, epigenetic changes, and environmental exposures [75, 76]. However, this model is increasingly being reconsidered in light of emerging evidence highlighting the role of the local microenvironment in shaping disease evolution.

In this context, periodontitis may contribute to carcinogenic processes by influencing the biological trajectory of pre-existing epithelial alterations [77, 78]. Rather than acting as an isolated trigger, periodontal disease may function as a contextual modifier capable of sustaining a permissive milieu in which dysplastic change is more likely to persist, expand, and acquire additional malignant features.

One of the key mechanisms through which periodontitis may contribute to malignant transformation is the persistence of a chronic inflammatory milieu. As discussed in previous sections, sustained inflammatory signaling can promote epithelial proliferation, inhibit apoptosis, and enhance angiogenesis, conditions that may facilitate the progression of dysplastic lesions [31, 36, 79]. In OPMDs, where epithelial homeostasis is already disrupted, the presence of periodontal inflammation may promote the accumulation of molecular alterations required for malignant transformation [52, 80, 81]. This is particularly relevant because the inflammatory microenvironment does not simply accompany epithelial change, but may actively influence clonal selection, tissue remodelling, and the survival advantage of altered cell populations. In this sense, periodontitis may amplify biological processes that are already operating within potentially malignant lesions, thereby lowering the threshold for malignant progression.

In parallel, periodontitis-associated microbial dysbiosis may further influence this transition. The presence of pathogenic species and their virulence factors can directly interact with epithelial cells, modulating signaling pathways involved in cell survival, immune evasion, and tissue invasion [13, 42, 82]. Moreover, microbial metabolites may exacerbate genomic instability and epigenetic dysregulation, thereby contributing to the progression of OPMDs towards OSCC [83]. Importantly, these effects are likely to be context-dependent and to interact with host susceptibility and other risk factors [84, 85]. This point deserves particular attention, as it suggests that dysbiosis is unlikely to act uniformly across all lesions, but may acquire greater pathogenic relevance in epithelial fields already primed by dysplasia, tobacco exposure, alcohol consumption, or impaired immune surveillance. Such a model may help explain why lesions with apparently similar histopathological features can follow markedly different clinical trajectories.

The concept of field cancerization provides an additional layer of interpretation. OPMDs do not develop in isolation but within a broader epithelial field that may already harbour molecular alterations. In this setting, periodontitis may contribute to the expansion or intensification of this altered field through both inflammatory and microbial mechanisms, thereby increasing the likelihood of malignant transformation [47, 48, 86]. This perspective is consistent with the emerging view of carcinogenesis as a spatially and temporally dynamic process shaped by continuous interactions between epithelial cells and their microenvironment [87, 88]. It also raises the possibility that periodontal inflammation may not only influence the index lesion itself, but also the surrounding mucosa, thereby contributing to a wider ecological and biological instability within the oral cavity. This broader spatial dimension is particularly relevant when considering multifocal disease, lesion recurrence, or the emergence of secondary lesions in patients with chronically altered oral environments.

Despite the biological plausibility of this model, direct clinical evidence remains limited. Most available studies have focused either on the association between periodontitis and OSCC or on the natural history of OPMDs, without specifically addressing the interaction between these conditions. Furthermore, existing data are predominantly derived from cross-sectional or retrospective designs, which limits causal inference. As a result, the potential role of periodontitis as a modifier of OPMD progression remains largely hypothetical, although increasingly supported by indirect evidence [89]. This limitation should not weaken the relevance of the hypothesis; rather, it underscores the need to move from associative observations to mechanism-informed clinical research capable of testing whether periodontal status meaningfully affects malignant risk over time.

From a clinical standpoint, recognising periodontitis as a potential co-factor in malignant transformation could have significant implications. Integrating periodontal assessment into the management of patients with OPMDs may contribute to a more comprehensive evaluation of risk. Moreover, periodontal therapy could, in theory, represent a modifiable factor capable of influencing the local microenvironment and potentially altering disease trajectory. However, these hypotheses require validation through well-designed longitudinal and interventional studies.

**Fig. 7:**
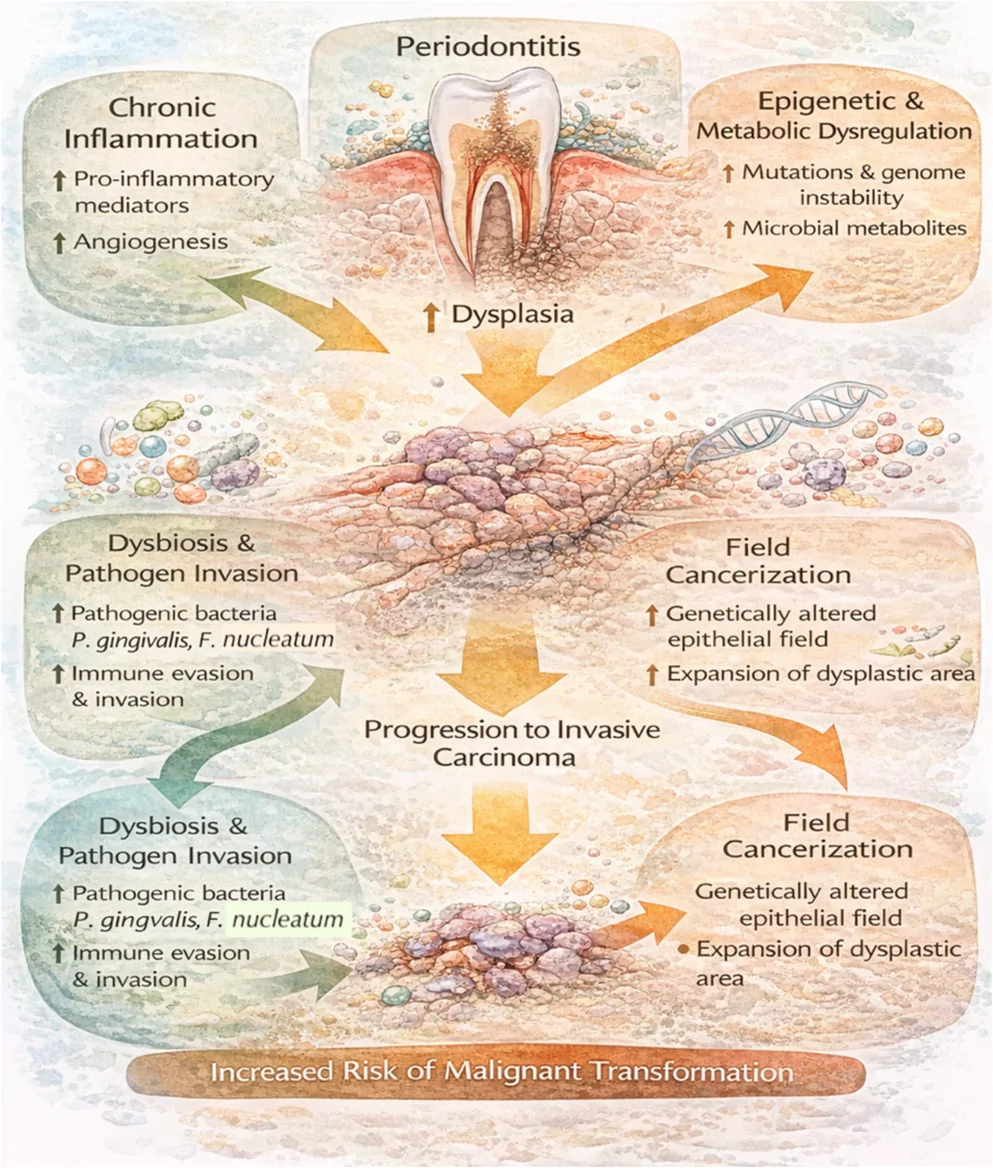
Schematic representation of how chronic odontogenic inflammation drives disease progression

Overall, the transition from OPMDs to OSCC represents a critical but still insufficiently investigated interface in which periodontitis may play a modulatory role. Addressing this gap may help transition towards a more integrated and clinically actionable model of oral carcinogenesis. From a clinical standpoint, this highlights a potentially actionable interface where modifying the local microenvironment may influence disease trajectory. The potential role of periodontitis across the transition from OPMDs to OSCC is schematically summarised in Fig. 7.

## Evidence Synthesis and Translational Implications

The evidence synthesised in this review supports the hypothesis that periodontitis may act as a contextual modifier in the malignant transformation of oral potentially malignant disorders (OPMDs). Rather than indicating a direct causal role, the available literature suggests that periodontal inflammation and dysbiosis may contribute to a biologically permissive oral environment in which early carcinogenic processes are more likely to persist or progress. This interpretation is supported by converging evidence from epidemiological studies on periodontitis and OSCC, microbiome studies comparing healthy mucosa, OPMDs, and OSCC, and mechanistic investigations on inflammation-mediated and microbiome-mediated carcinogenesis [48, 59, 90].

A central point emerging from the current evidence is the need to distinguish between different levels of support. The association between periodontitis and established OSCC is increasingly documented by observational studies and meta-analytic data, although residual confounding remains an important limitation. By contrast, the specific role of periodontitis in the transition from OPMDs to invasive carcinoma remains much less clearly demonstrated [52, 91].

Evidence regarding this intermediate phase is still largely indirect and derives mainly from biological plausibility, translational studies, and observations of microbial and inflammatory alterations across the spectrum of oral carcinogenesis. This distinction is clinically relevant, because the OPMD stage represents the phase in which surveillance, risk stratification, and preventive interventions may have the greatest potential impact.

From a mechanistic perspective, periodontitis may influence early oral carcinogenesis through several interconnected pathways. Chronic inflammatory signalling may promote epithelial proliferation, survival, angiogenesis, and tissue remodelling, while reactive oxygen and nitrogen species may contribute to DNA damage, genomic instability, and epigenetic dysregulation [92].

In parallel, periodontal pathogens such as Porphyromonas gingivalis and Fusobacterium nucleatum may modulate epithelial signalling, immune surveillance, invasion-related pathways, and microbiome-associated metabolic reprogramming. These mechanisms are biologically compelling, but most supporting data derive from experimental, translational, or OSCC-based settings rather than from longitudinal studies specifically designed to assess OPMD progression.

Within this framework, field cancerization offers a useful interpretative model. OPMDs do not develop in isolation, but within a broader mucosal field that may already be genetically, epigenetically, or biologically altered. Periodontitis may contribute to this field by sustaining inflammatory mediator release, microbial exposure, oxidative stress, and epithelial barrier disruption beyond the periodontal niche itself [52, 76]. In this perspective, periodontal disease may not initiate malignant transformation independently, but may intensify or maintain a local environment that favours clonal persistence, epithelial instability, and progression of pre-existing dysplastic changes.

The microbiome represents another relevant component of this model. Evidence showing compositional and functional differences in the oral microbiome across healthy mucosa, OPMDs, and OSCC supports the hypothesis of a progressive ecological shift during oral carcinogenesis [74, 91]. However, the directionality of this relationship remains unresolved. Dysbiosis may contribute to malignant progression, but it may also arise as a consequence of epithelial alteration, immune remodelling, changes in oral hygiene, smoking exposure, lesion ulceration, or tumour-related environmental changes. Therefore, microbiome findings should be interpreted cautiously and should not be assumed to indicate causality.

Methodological heterogeneity represents a major limitation of the current literature. Most studies are cross-sectional or retrospective, limiting causal inference and making it difficult to determine whether periodontal inflammation and dysbiosis are drivers of progression, consequences of epithelial change, or both. In addition, variability in periodontal definitions, OPMD classification, microbiome sampling sites, sequencing platforms, analytical pipelines, and adjustment for confounders complicates comparisons across studies. Shared risk factors, including tobacco use, alcohol consumption, age, socioeconomic status, oral hygiene, and systemic inflammatory conditions, remain particularly important sources of residual confounding.

The heterogeneity of available evidence and its main limitations are summarized in Table 2.

**Table 2:** Summary of current evidence linking periodontitis, microbiome alterations, and oral carcinogenesis

Topic	Type of evidence	Key findings	Strengths	Limitations
Periodontitis–OSCC association	Epidemiological studies and meta-analyses	Recurrent association between periodontal disease and OSCC risk	Consistency across several observational datasets	Residual confounding and limited causal inference
Oral microbiome alterations	Molecular and translational studies	Dysbiosis associated with OPMDs and OSCC, with compositional and functional shifts	Mechanistic plausibility	Heterogeneity in sampling and analytical methods
OPMDs progression	Limited clinical evidence	Possible contribution of inflammation and dysbiosis to malignant transformation	Emerging biologically informed framework	Lack of longitudinal studies
Microbial metabolites	Experimental and translational studies	Metabolic by-products may influence epithelial integrity, immunity, and genomic stability	Strong biological rationale	Limited direct clinical validation

From a translational standpoint, the hypothesis that periodontitis may contribute to a permissive microenvironment for malignant transformation is relevant, but it should not yet be translated into claims of established prognostic value. Periodontal status should not currently be considered an independent risk marker comparable to dysplasia grade, lesion subtype, anatomical site, or other validated predictors of malignant transformation. Nevertheless, periodontal assessment may reasonably be incorporated into a comprehensive oral evaluation of patients with OPMDs, particularly because periodontal inflammation represents a modifiable condition and may contribute to the overall inflammatory and microbial burden of the oral cavity [47, 89].

Similarly, the potential role of periodontal therapy should be interpreted with caution. No direct evidence currently demonstrates that periodontal treatment reduces the risk of malignant transformation in OPMDs. However, periodontal therapy may reduce local inflammatory burden and partially restore microbial balance, making it a biologically plausible strategy for modifying the oral microenvironment. At present, this should be viewed as a hypothesis-generating concept rather than an evidence-based preventive intervention.

Future research should move beyond general associations and address clinically testable questions. Prospective longitudinal studies are needed to determine whether periodontitis independently predicts malignant transformation in patients with OPMDs after adjustment for established risk factors. Translational studies integrating periodontal parameters, histopathology, microbiome profiling, inflammatory biomarkers, and clinical outcomes could help identify microbial or immune signatures associated with high-risk lesions. Finally, interventional studies are required to determine whether periodontal therapy can modify local inflammatory and microbial patterns in ways that are biologically meaningful for OPMD evolution.

Overall, the present synthesis supports an integrated model of oral carcinogenesis in which epithelial alterations, periodontal inflammation, microbial dysbiosis, metabolic activity, and field effects interact dynamically. Within this model, periodontitis should be regarded less as a direct cause of malignant transformation and more as a potential contextual modifier of early carcinogenic processes. Clarifying this relationship may improve biological understanding and, in the future, contribute to more refined risk stratification and more comprehensive management of patients with OPMDs.

## Clinical Relevance for OPMD Surveillance

Although current evidence is insufficient to support formal changes in clinical guidelines, the potential relationship between periodontitis and OPMD progression may have practical relevance for patient assessment and interdisciplinary management. In particular, periodontal status should not be considered an established independent predictor of malignant transformation, but it may represent an additional clinical variable within a comprehensive evaluation of patients with OPMDs.

In this context, periodontal assessment may be incorporated into routine oral examination, in line with broader clinical approaches integrating local inflammatory and mucosal conditions in oral lesion assessment [12, 93]. Parameters such as probing depth, clinical attachment loss, bleeding on probing, plaque control, and signs of active inflammation may help clinicians characterise the broader oral environment in which OPMDs occur. This approach does not replace conventional risk assessment based on lesion subtype, anatomical site, clinical behaviour, and histopathological grading, but may complement it by considering modifiable inflammatory and microbial factors.

The management of periodontal inflammation may also be clinically relevant, although its impact on malignant transformation remains unproven. Non-surgical periodontal therapy and supportive periodontal care can reduce local inflammatory burden and improve periodontal stability. Therefore, treating active periodontitis in patients with OPMDs may be justified as part of comprehensive oral care, while its potential role in modifying carcinogenic risk should currently be regarded as biologically plausible but not clinically demonstrated.

These considerations support an interdisciplinary approach involving oral medicine specialists, periodontists, and pathologists. Such collaboration may allow a more integrated assessment of epithelial lesions and the surrounding periodontal condition, particularly in patients requiring long-term surveillance. Patient education should also remain central, including reinforcement of oral hygiene, regular periodontal maintenance, and control of established risk factors such as tobacco and alcohol use.

In practical terms, clinicians managing patients with OPMDs may consider:

(i) routine periodontal assessment at baseline;
(ii) early management of periodontal inflammation in patients with dysplastic lesions;
(iii) closer follow-up in the presence of active periodontal disease;
(iv) interdisciplinary collaboration between oral medicine specialists and periodontists.

Although not yet formalised in clinical guidelines, these strategies are consistent with current biological evidence and may contribute to a more comprehensive and risk-oriented management approach.

## Limitations of Current Evidence

Despite the growing body of literature supporting a potential link between periodontitis and oral carcinogenesis, several limitations should be acknowledged, particularly regarding the role of periodontal disease in the progression from oral potentially malignant disorders (OPMDs) to oral squamous cell carcinoma (OSCC).

First, direct evidence on the interaction between periodontitis and OPMD progression remains limited. Most available studies have focused either on the association between periodontal disease and established OSCC or on the natural history of OPMDs, considered independently. As a result, the hypothesis that periodontitis may act as a modulator of malignant transformation is still supported mainly by indirect biological, epidemiological, and translational evidence rather than by dedicated clinical studies.

Second, much of the current evidence derives from observational, cross-sectional, or retrospective studies, which limits causal inference. This is particularly relevant because periodontitis and oral cancer share several risk determinants, including smoking, alcohol consumption, socioeconomic status, oral hygiene practices, and systemic inflammatory conditions. Although some studies adjust for these variables, residual confounding cannot be excluded.

Third, methodological heterogeneity remains substantial. Variability in periodontal definitions, OPMD classification, clinical endpoints, microbiome sampling procedures, sequencing techniques, and analytical methods complicates comparisons across studies and may contribute to inconsistent findings. This heterogeneity also limits the reproducibility and generalisability of microbiological and mechanistic observations.

A further limitation is the lack of longitudinal and interventional studies. At present, there is insufficient prospective evidence to demonstrate whether periodontitis influences the rate of malignant transformation in OPMDs or whether periodontal treatment can modify this risk. This gap limits the translation of current biological plausibility into evidence-based clinical recommendations.

Finally, oral carcinogenesis is a complex and multifactorial process involving genetic susceptibility, environmental exposures, host immune responses, microbial ecology, and local tissue factors. Isolating the specific contribution of periodontitis within this broader framework remains challenging, and there is a risk of overestimating its role if findings are not interpreted cautiously.

Taken together, these limitations highlight the need for prospective studies using standardised periodontal and OPMD definitions, adequate adjustment for shared risk factors, and integrative approaches combining clinical, histopathological, microbiological, and molecular data. Addressing these gaps will be essential to clarify whether periodontitis has a clinically meaningful role in OPMD progression.

## Conclusions

The relationship between periodontitis and oral carcinogenesis is an emerging area of clinical and biological interest. Although the association between periodontal disease and established OSCC is increasingly recognised, its role in the transition from OPMDs to invasive carcinoma remains insufficiently defined. Current evidence suggests that chronic inflammation, microbial dysbiosis, metabolic alterations, and field cancerization may create a permissive oral environment in which periodontitis could act as a contextual modifier of early carcinogenic processes. However, the available evidence remains largely indirect and does not establish causality or prognostic value. From a clinical perspective, periodontal evaluation may complement conventional assessment of patients with OPMDs, but periodontal status should not yet be considered an independent predictor of malignant transformation. Future longitudinal and interventional studies are needed to clarify whether periodontitis meaningfully influences OPMD progression and whether periodontal therapy can modify disease trajectory.

## Key References

- Pigossi SC, Oliveira JA, de Medeiros MC, Soares LFF, D’Silva NJ. Demystifying the link between periodontitis and oral cancer: a systematic review integrating clinical, pre-clinical, and in vitro data. Cancer Metastasis Rev. 2025;44:67. 10.1007/s10555-025-10285-z.
  - ◦ This systematic review integrates clinical, pre-clinical, and in vitro evidence on the association between periodontitis and oral cancer.
- Baima G, Minoli M, Michaud DS, Aimetti M, Sanz M, Loos BG, et al. Periodontitis and risk of cancer: Mechanistic evidence. Periodontol 2000. 2024;96:83–94. 10.1111/prd.12540.
  - ◦ This review provides a comprehensive mechanistic framework linking periodontitis to cancer development.
- Yuan Q, Wu H, Tan H, Wang X, Cao Y, Chen G. Oral microbial dysbiosis driven by periodontitis facilitates oral squamous cell carcinoma progression. Cancers (Basel). 2025;17:2181. 10.3390/cancers17132181.
  - ◦ This experimental study provides evidence that periodontitis-associated dysbiosis may facilitate OSCC progression.
- Pignatelli P, Curia MC, Tenore G, Bondi D, Piattelli A, Romeo U. Oral bacteriome and oral potentially malignant disorders: A systematic review of the associations. Arch Oral Biol. 2024;160:105891. 10.1016/j.archoralbio.2024.105891.
  - ◦ This systematic review summarizes current evidence on oral bacteriome alterations in OPMDs.
- Liu Y, Wu W, Liang Q, Diao J, Yang W, Zheng S, et al. Metabolic and microbial alterations in oral potentially malignant disorders versus oral squamous cell carcinoma. Sci Rep. 2025;15:40637. 10.1038/s41598-025-24363-3.
  - ◦ This study identifies microbial and metabolic differences between OPMDs and OSCC, supporting their potential role in malignant progression.
- Wu Y, Han B, Zhang B, Li J, Ren B, Su Z. Metabolic crosstalk between oral microbiota and the host in OSCC: emerging roles of microbial metabolites in tumor initiation and progression. Front Cell Infect Microbiol. 2026;16:1778329. 10.3389/fcimb.2026.1778329.
  - ◦ This review highlights the role of microbiota-derived metabolites in OSCC initiation and progression.

## Data Availability

No datasets were generated or analysed during the current study.

## References

[1] Warnakulasuriya S. Oral potentially malignant disorders: A comprehensive review on clinical aspects and management. Oral Oncol. 2020;102:104550. 31981993 10.1016/j.oraloncology.2019.104550

[2] Speight PM, Khurram SA, Kujan O. Oral potentially malignant disorders: risk of progression to malignancy. Oral Surg Oral Med Oral Pathol Oral Radiol. 2018;125(6):612–27. 29396319 10.1016/j.oooo.2017.12.011

[3] Kujan O, Oliver RJ, Khattab A, Roberts SA, Thakker N, Sloan P. Evaluation of a new binary system of grading oral epithelial dysplasia for prediction of malignant transformation. Oral Oncol. 2006;42(10):987–93. 16731030 10.1016/j.oraloncology.2005.12.014

[4] Villa A, Woo SB. Leukoplakia-A Diagnostic and Management Algorithm. J Oral Maxillofac Surg. 2017;75(4):723–34. 27865803 10.1016/j.joms.2016.10.012

[5] Kujan O. Oral Potentially Malignant Disorders: Current Knowledge and Future Directions. Dent Clin North Am. 2025;69(3):327–46. 40545327 10.1016/j.cden.2025.03.001

[6] Kinane DF, Stathopoulou PG, Papapanou PN. Periodontal diseases. Nat Rev Dis Primers. 2017;3:17038. 28805207 10.1038/nrdp.2017.38

[7] Coussens LM, Werb Z. Inflammation and cancer. Nature. Dec. 2002;19–26(6917):860–7. 10.1038/nature01322 PMC2803035 12490959

[8] Tezal M. Interaction between Chronic Inflammation and Oral HPV Infection in the Etiology of Head and Neck Cancers. Int J Otolaryngol. 2012;2012:575242. 22518158 10.1155/2012/575242 PMC3299260

[9] Perera M, Al-Hebshi NN, Speicher DJ, Perera I, Johnson NW. Emerging role of bacteria in oral carcinogenesis: a review with special reference to perio-pathogenic bacteria. J Oral Microbiol. 2016;8:32762. 27677454 10.3402/jom.v8.32762 PMC5039235

[10] Corbella S, Veronesi P, Galimberti V, Weinstein R, Del Fabbro M, Francetti L. Is periodontitis a risk indicator for cancer? A meta-analysis. PLoS ONE. 2018;13(4):e0195683. 29664916 10.1371/journal.pone.0195683 PMC5903629

[11] Michaud DS, Fu Z, Shi J, Chung M. Periodontal Disease, Tooth Loss, and Cancer Risk. Epidemiol Rev. 2017;39(1):49–58. 28449041 10.1093/epirev/mxx006 PMC5868279

[12] Whitmore SE, Lamont RJ. Oral bacteria and cancer. PLoS Pathog. 2014;10(3):e1003933. 24676390 10.1371/journal.ppat.1003933 PMC3968118

[13] Gao S, Li S, Ma Z, Liang S, Shan T, Zhang M, Zhu X, Zhang P, Liu G, Zhou F, Yuan X, Jia R, Potempa J, Scott DA, Lamont RJ, Wang H, Feng X. Presence of Porphyromonas gingivalis in esophagus and its association with the clinicopathological characteristics and survival in patients with esophageal cancer. Infect Agent Cancer. 2016;11:3. 26788120 10.1186/s13027-016-0049-x PMC4717526

[14] Rubinstein MR, Wang X, Liu W, Hao Y, Cai G, Han YW. Fusobacterium nucleatum promotes colorectal carcinogenesis by modulating E-cadherin/β-catenin signaling via its FadA adhesin. Cell Host Microbe. 2013;14(2):195–206. 23954158 10.1016/j.chom.2013.07.012 PMC3770529

[15] Olsen I, Yilmaz Ö. Modulation of inflammasome activity by Porphyromonas gingivalis in periodontitis and associated systemic diseases. J Oral Microbiol. 2016;8:30385. 26850450 10.3402/jom.v8.30385 PMC4744328

[16] Brennan CA, Garrett WS. Fusobacterium nucleatum - symbiont, opportunist and oncobacterium. Nat Rev Microbiol. 2019;17(3):156–66. 30546113 10.1038/s41579-018-0129-6 PMC6589823

[17] Grivennikov SI, Greten FR, Karin M. Immunity, inflammation, and cancer. Cell. 2010;140(6):883–99. 20303878 10.1016/j.cell.2010.01.025 PMC2866629

[18] Reuter S, Gupta SC, Chaturvedi MM, Aggarwal BB. Oxidative stress, inflammation, and cancer: how are they linked? Free Radic Biol Med. 2010;49(11):1603–16. 20840865 10.1016/j.freeradbiomed.2010.09.006 PMC2990475

[19] Schmidt BL, Kuczynski J, Bhattacharya A, Huey B, Corby PM, Queiroz EL, Nightingale K, Kerr AR, DeLacure MD, Veeramachaneni R, Olshen AB, Albertson DG. Changes in abundance of oral microbiota associated with oral cancer. PLoS ONE. 2014;9(6):e98741. 24887397 10.1371/journal.pone.0098741 PMC4041887

[20] Zhao H, Chu M, Huang Z, Yang X, Ran S, Hu B, Zhang C, Liang J. Variations in oral microbiota associated with oral cancer. Sci Rep. 2017;7(1):11773. 28924229 10.1038/s41598-017-11779-9 PMC5603520

[21] Herreros-Pomares A, Hervás D, Bagan-Debón L, Jantus-Lewintre E, Gimeno-Cardona C, Bagan J. On the Oral Microbiome of Oral Potentially Malignant and Malignant Disorders: Dysbiosis, Loss of Diversity, and Pathogens Enrichment. Int J Mol Sci. 2023;24:3466. 36834903 10.3390/ijms24043466 PMC9961214

[22] Zhang L, Liu Y, Zheng HJ, Zhang CP. The Oral Microbiota May Have Influence on Oral Cancer. Front Cell Infect Microbiol. 2020;9:476. 32010645 10.3389/fcimb.2019.00476 PMC6974454

[23] Mok SF, Karuthan C, Cheah YK, Ngeow WC, Rosnah Z, Yap SF, Ong HKA. The oral microbiome community variations associated with normal, potentially malignant disorders and malignant lesions of the oral cavity. Malays J Pathol. 2017;39(1):1–15. 28413200

[24] Slaughter DP, Southwick HW, Smejkal W. Field cancerization in oral stratified squamous epithelium; clinical implications of multicentric origin. Cancer. 1953;6(5):963–8. 13094644 10.1002/1097-0142(195309)6:5<963::aid-cncr2820060515>3.0.co;2-q

[25] Braakhuis BJ, Tabor MP, Kummer JA, Leemans CR, Brakenhoff RH. A genetic explanation of Slaughter’s concept of field cancerization: evidence and clinical implications. Cancer Res. 2003;63(8):1727–30. PMID: 12702551. 12702551

[26] Roi A, Ioan Roi C, Rivis M, Luca R, Boia S, Rusu L-C. Periodontal Pathogens and their Involvement in the Oral Carcinogenesis Process [Internet]. Periodontal Frontiers [Working Title]. IntechOpen; 2025.

[27] Greten FR, Grivennikov SI. Inflammation and Cancer: Triggers, Mechanisms, and Consequences. Immunity. 2019;51(1):27–41. 31315034 10.1016/j.immuni.2019.06.025 PMC6831096

[28] Kitamura T, Qian BZ, Pollard JW. Immune cell promotion of metastasis. Nat Rev Immunol. 2015;15(2):73–86. 25614318 10.1038/nri3789 PMC4470277

[29] Tonetti MS, Greenwell H, Kornman KS. Staging and grading of periodontitis: Framework and proposal of a new classification and case definition. J Periodontol. 2018;89(Suppl 1):S159–72. 29926952 10.1002/JPER.18-0006

[30] Van Dyke TE, Bartold PM, Reynolds EC. The Nexus Between Periodontal Inflammation and Dysbiosis. Front Immunol. 2020;11:511. 32296429 10.3389/fimmu.2020.00511 PMC7136396

[31] Irani S, Barati I, Badiei M. Periodontitis and oral cancer - current concepts of the etiopathogenesis. Oncol Rev. 2020;14(1):465. 32231765 10.4081/oncol.2020.465 PMC7097927

[32] Ramadan DE, Hariyani N, Indrawati R, Ridwan RD, Diyatri I. Cytokines and Chemokines in Periodontitis. Eur J Dent. 2020;14(3):483–95. 32575137 10.1055/s-0040-1712718 PMC7440949

[33] Båge T, Kats A, Lopez BS, Morgan G, Nilsson G, Burt I, Korotkova M, Corbett L, Knox AJ, Pino L, Jakobsson PJ, Modéer T, Yucel-Lindberg T. Expression of prostaglandin E synthases in periodontitis immunolocalization and cellular regulation. Am J Pathol. 2011;178(4):1676–88. 21435451 10.1016/j.ajpath.2010.12.048 PMC3078457

[34] Landskron G, De la Fuente M, Thuwajit P, Thuwajit C, Hermoso MA. Chronic inflammation and cytokines in the tumor microenvironment. J Immunol Res. 2014;2014:149185. 24901008 10.1155/2014/149185 PMC4036716

[35] Laha D, Grant R, Mishra P, Nilubol N. The Role of Tumor Necrosis Factor in Manipulating the Immunological Response of Tumor Microenvironment. Front Immunol. 2021;12:656908. 33986746 10.3389/fimmu.2021.656908 PMC8110933

[36] Niklander SE. Inflammatory Mediators in Oral Cancer: Pathogenic Mechanisms and Diagnostic Potential. Front Oral Health. 2021;2:642238. 35047997 10.3389/froh.2021.642238 PMC8757707

[37] Nishida A, Andoh A. The Role of Inflammation in Cancer: Mechanisms of Tumor Initiation, Progression, and Metastasis. Cells. 2025;14(7):488. 40214442 10.3390/cells14070488 PMC11987742

[38] Deng S, Wang S, Shi X, Zhou H. Microenvironment in Oral Potentially Malignant Disorders: Multi-Dimensional Characteristics and Mechanisms of Carcinogenesis. Int J Mol Sci. 2022;23(16):8940. 36012205 10.3390/ijms23168940 PMC9409092

[39] Dionigi C, Larsson L, Carcuac O, Berglundh T. Cellular expression of DNA damage/repair and reactive oxygen/nitrogen species in human periodontitis and peri-implantitis lesions. J Clin Periodontol. 2020;47(12):1466–75. 32996143 10.1111/jcpe.13370 PMC7756411

[40] Bullon P, Giampieri F, Bullon B, Battino M. The role of oxidative stress in periodontitis. J Periodontal Res. 2025. 10.1111/jre.70016 10.1111/jre.70016 40671662

[41] Rembiałkowska N, Kocik Z, Kłosińska A, Kübler M, Pałkiewicz A, Rozmus W, Sędzik M, Wojciechowska H, Gajewska-Naryniecka A. Inflammation-Driven Genomic Instability: A Pathway to Cancer Development and Therapy Resistance. Pharmaceuticals (Basel). 2025;18(9):1406. 41011273 10.3390/ph18091406 PMC12472738

[42] Wang B, Deng J, Donati V, Merali N, Frampton AE, Giovannetti E, Deng D. The Roles and Interactions of *Porphyromonas gingivalis* and *Fusobacterium nucleatum* in Oral and Gastrointestinal Carcinogenesis: A Narrative Review. Pathogens. 2024;13(1):93. 38276166 10.3390/pathogens13010093 PMC10820765

[43] Liu S, Zhou X, Peng X, Li M, Ren B, Cheng G, Cheng L. *Porphyromonas gingivalis* Promotes Immunoevasion of Oral Cancer by Protecting Cancer from Macrophage Attack. J Immunol. 2020;205(1):282–9. 32471882 10.4049/jimmunol.1901138

[44] McIlvanna E, Linden GJ, Craig SG, Lundy FT, James JA. Fusobacterium nucleatum and oral cancer: a critical review. BMC Cancer. 2021;21(1):1212. 34774023 10.1186/s12885-021-08903-4 PMC8590362

[45] Semiz V, Aksoy RA, Altun Z. Oral Microbiome-Mediated Carcinogenesis in Oral Cavity Cancer: A Narrative Review. Cureus. 2025;17(4):e83242. 40453286 10.7759/cureus.83242 PMC12123651

[46] Sukmana BI, Saleh RO, Najim MA, AL-Ghamdi HS, Achmad H, Al-Hamdani MM, Taher AAY, Alsalamy A, Khaledi M, Javadi K. Oral microbiota and oral squamous cell carcinoma: a review of their relation and carcinogenic mechanisms. Front Oncol. 2024;14:1319777. 38375155 10.3389/fonc.2024.1319777 PMC10876296

[47] Bonilla M, Peñalver I, Mesa-López MJ, Mesa F. Association Between Periodontitis and Cancer: A Perspective Review of Mechanisms and Clinical Evidence. J Clin Med. 2025;14(17):6334. 40944094 10.3390/jcm14176334 PMC12428835

[48] Baima G, Minoli M, Michaud DS, Aimetti M, Sanz M, Loos BG, Romandini M. Periodontitis and risk of cancer: Mechanistic evidence. Periodontol 2000. 2024;96(1):83–94. 38102837 10.1111/prd.12540 PMC11579815

[49] Santacroce L, Jirillo E, Bottalico L, Topi S, Passarelli PC, Mancini A, D’Addona A, Garcia Godoy F, Folliero V, Colella M. Does a link exist between oral microbiota and oral squamous cell carcinoma? A review of current insights. J Oral Microbiol. 2025;17(1):2569934. 41221327 10.1080/20002297.2025.2569934 PMC12599010

[50] Mohan M, Jagannathan N. Oral field cancerization: an update on current concepts. Oncol Rev. 2014;8(1):244. 25992232 10.4081/oncol.2014.244 PMC4419611

[51] Kurago Z, Loveless J. Microbial Colonization and Inflammation as Potential Contributors to the Lack of Therapeutic Success in Oral Squamous Cell Carcinoma. Front Oral Health. 2021;2:739499. 35048056 10.3389/froh.2021.739499 PMC8757816

[52] Ali A, Molska GR, Yeo H, Esfandiari N, Jeong W, Huang M, Magalhaes M. Immune Microenvironment in Oral Potentially Malignant Disorders and Oral Cancer: A Narrative Review. Int J Mol Sci. 2025;26(14):6650. 40724900 10.3390/ijms26146650 PMC12294717

[53] Hajishengallis G, Chavakis T. Local and systemic mechanisms linking periodontal disease and inflammatory comorbidities. Nat Rev Immunol. 2021;21(7):426–40. 33510490 10.1038/s41577-020-00488-6 PMC7841384

[54] Yang S, Yang X. The Role of Reactive Oxygen Species (ROS) in Periodontitis: A Potential Therapeutic Target. Immun Inflamm Dis. 2025;13(11):e70301. 41298339 10.1002/iid3.70301 PMC12657125

[55] Lin D, Yang L, Wen L, Lu H, Chen Q, Wang Z. Crosstalk between the oral microbiota, mucosal immunity, and the epithelial barrier regulates oral mucosal disease pathogenesis. Mucosal Immunol. 2021;14(6):1247–58. 34040155 10.1038/s41385-021-00413-7

[56] Hajishengallis G, Lamont RJ, Koo H. Oral polymicrobial communities: Assembly, function, and impact on diseases. Cell Host Microbe. 2023;31(4):528–38. 36933557 10.1016/j.chom.2023.02.009 PMC10101935

[57] Urzică R-N, Crețu B, Căruntu A, Bucurica S, Farcasiu A-T, Ciupescu LM, Scheau C, Căruntu C. The Molecular Interplay Between Oral Microbiome and Oral Cancer Pathogenesis. Int J Mol Sci. 2025;26:10212. 41155504 10.3390/ijms262010212 PMC12564225

[58] Liu Y, Wu W, Liang Q, et al. Metabolic and microbial alterations in oral potentially malignant disorders versus oral squamous cell carcinoma. Sci Rep. 2025;15:40637. 41254023 10.1038/s41598-025-24363-3 PMC12627484

[59] Pignatelli P, Curia MC, Tenore G, Bondi D, Piattelli A, Romeo U. Oral bacteriome and oral potentially malignant disorders: A systematic review of the associations. Arch Oral Biol. 2024;160:105891. 38295615 10.1016/j.archoralbio.2024.105891

[60] Devaraja K, Aggarwal S. Dysbiosis of Oral Microbiome: A Key Player in Oral Carcinogenesis? Crit Rev Biomedicines. 2025;13(2):448. 10.3390/biomedicines13020448 PMC11852717 40002861

[61] Intini R, Balsells S, Bagan L, Fortuna G, Sroussi H, Bagan J. Comparative analysis of oral microbiome in saliva samples of oral leukoplakia, proliferative leukoplakia and oral squamous cell carcinoma. Front Oral Health. 2025;6:1600090. 40438084 10.3389/froh.2025.1600090 PMC12116504

[62] Lan Q, Zhang C, Hua H, et al. Compositional and functional changes in the salivary microbiota related to oral leukoplakia and oral squamous cell carcinoma: a case control study. BMC Oral Health. 2023;23:1021. 38115005 10.1186/s12903-023-03760-y PMC10731685

[63] Deo PN, Deshmukh RS, Gaike AH, Christopher A, Gujare M, Inamdar M. Oral microbiome profiles in oral potentially malignant disorders and oral cancer - A diagnostic perspective. J Oral Maxillofac Pathol. 2025;29(1):87–97. 40248614 10.4103/jomfp.jomfp_140_24 PMC12002586

[64] Lamont RJ, Fitzsimonds ZR, Wang H, Gao S. Role of Porphyromonas gingivalis in oral and orodigestive squamous cell carcinoma. Periodontol 2000. 2022;89(1):154–65. 35244980 10.1111/prd.12425 PMC9439709

[65] Galasso L, Termite F, Mignini I, Esposto G, Borriello R, Vitale F, Nicoletti A, Paratore M, Ainora ME, Gasbarrini A, et al. Unraveling the Role of *Fusobacterium nucleatum* in Colorectal Cancer: Molecular Mechanisms and Pathogenic Insights. Cancers. 2025;17:368. 39941737 10.3390/cancers17030368 PMC11816155

[66] Song X, Wang J, Gu Z, et al. *Porphyromonas gingivalis* and *Fusobacterium nucleatum* synergistically strengthen the effect of promoting oral squamous cell carcinoma progression. Infect Agents Cancer. 2025;20:60. 10.1186/s13027-025-00689-5 PMC12398151 40883778

[67] Pérez Escriva P, Correia Tavares Bernardino C, Letellier E. De-coding the complex role of microbial metabolites in cancer. Cell Rep. 2025;44(3):115358. 40023841 10.1016/j.celrep.2025.115358

[68] Lei J, Wang X, Liu X. Microbiota-derived metabolites in the epigenetic regulation of the host. Sci Bull (Beijing). 2025;70(21):3667–78. 41047314 10.1016/j.scib.2025.09.030

[69] Wu Y, Han B, Zhang B, Li J, Ren B, Su Z. Metabolic crosstalk between oral microbiota and the host in OSCC: emerging roles of microbial metabolites in tumor initiation and progression. Front Cell Infect Microbiol. 2026;16:1778329. 41769336 10.3389/fcimb.2026.1778329 PMC12945793

[70] Menghani SV. Carcinogenetic mechanisms employed by the oral microbiome: A narrative review. Am J Med Sci. 2025;369(5):556–61. 39788425 10.1016/j.amjms.2025.01.001

[71] Pratap Singh R, Kumari N, Gupta S, Jaiswal R, Mehrotra D, Singh S, Mukherjee S, Kumar R. Intratumoral Microbiota Changes with Tumor Stage and Influences the Immune Signature of Oral Squamous Cell Carcinoma. Microbiol Spectr. 2023;11(4):e0459622. 37409975 10.1128/spectrum.04596-22 PMC10434029

[72] Shen X, Zhang YL, Zhu JF, Xu BH. Oral dysbiosis in the onset and carcinogenesis of oral epithelial dysplasia: A systematic review. Arch Oral Biol. 2023;147:105630. 36709626 10.1016/j.archoralbio.2023.105630

[73] Yuan Q, Wu H, Tan H, Wang X, Cao Y, Chen G. Oral Microbial Dysbiosis Driven by Periodontitis Facilitates Oral Squamous Cell Carcinoma Progression. Cancers (Basel). 2025;17(13):2181. 40647479 10.3390/cancers17132181 PMC12249316

[74] Crispino A, Varricchio S, Esposito A, Marfella A, Cerbone D, Perna A, Petronio Petronio G, Staibano S, Merolla F, Ilardi G. The oral microbiome and its role in oral squamous cell carcinoma: a systematic review of microbial alterations and potential biomarkers. Pathologica. 2024;116(6):338–57. 39748720 10.32074/1591-951X-N867

[75] Prostakishina EA, Sidenko EA, Kolegova ES, Patysheva MR, Kononova GA, Choinzonov EL. Premalignant lesions of the oral cavity: a narrative review of factors and mechanisms of transformation into cancer. Int J Oral Maxillofac Surg. 2025;54(6):479–93. 39730281 10.1016/j.ijom.2024.12.006

[76] Wils LJ, Brakenhoff RH. Oral Potentially Malignant Disorders, an Update. In: Vermorken JB, Langendijk JA, Leemans CR, Machiels JP, Nicolai P, O’Sullivan B, editors. Critical Issues in Head and Neck Oncology. Cham: Springer; 2025.

[77] Sutera S, Furchì OA, Pentenero M. Macrophages and the immune microenvironment in OPMDs: a systematic review of the literature. Front Oral Health. 2025;6:1605978. 40432828 10.3389/froh.2025.1605978 PMC12106459

[78] Dieterle MP, Husari A, Steinberg T, Wang X, Ramminger I, Tomakidi P. Role of Mechanotransduction in Periodontal Homeostasis and Disease. J Dent Res. 2021;100(11):1210–9. 33870741 10.1177/00220345211007855

[79] Julián F, Beltran SM, Viafara-Garcia AP, Labrador J, Basterrechea. The Role of Periodontopathogens and Oral Microbiome in the Progression of Oral Cancer. A Review. Open Dentistry J. 2021;15:367–76.

[80] Osorio-Osorno YA, Parada-Sanchez MT. Oral keratinocyte-mediated inflammation and epithelial disruption: A narrative review on IRF6 signaling and oral carcinogenic risk. Int J Immunopathol Pharmacol. 2026;40:3946320251411432. 41492714 10.1177/03946320251411432 PMC12775295

[81] Munzone M, Marmo GM, Polizzi A, Marya A, Blasi A, Isola G. Periodontal inflammation as a negative stimulus for oral cancerization: the hidden role of periodontitis in oral cancerization. Oncologie. 2025;27(5):659–72.

[82] Liao X, Si H, Lai Y, Zhang X, Feng Y, Zhou T, Feng Y, Yu L. *Porphyromonas gingivalis*-OMVs promote the epithelial-mesenchymal transition of oral squamous cell carcinoma by inhibiting ferroptosis through the NF-κB pathway. J Oral Microbiol. 2025;17(1):2482924. 40206095 10.1080/20002297.2025.2482924 PMC11980236

[83] Mignogna MD, Fedele S, Lo Russo L, Lo Muzio L, Bucci E. Immune activation and chronic inflammation as the cause of malignancy in oral lichen planus: is there any evidence ? Oral Oncol. 2004;40(2):120–30. 14693234 10.1016/j.oraloncology.2003.08.001

[84] León-Letelier RA, Dou R, Vykoukal J, Yip-Schneider MT, Maitra A, Irajizad E, Wu R, Dennison JB, Do KA, Zhang J, Schmidt CM, Hanash S, Fahrmann JF. Contributions of the Microbiome-Derived Metabolome for Risk Assessment and Prognostication of Pancreatic Cancer. Clin Chem. 2024;70(1):102–15. 38175578 10.1093/clinchem/hvad186 PMC11836914

[85] Di Bello A, Scala A, Gili R, Falcicchia R, Bassolino A, Polidori S, D’Auria F, Tortora G, Bossi P, Cassano A. The microbiome in head and neck squamous cell carcinoma: Biological drivers, therapeutic interactions, and emerging clinical applications. Crit Rev Oncol Hematol. 2026;218:105108. 41485632 10.1016/j.critrevonc.2025.105108

[86] Partridge M, Emilion G, Pateromichelakis S, Phillips E, Langdon J. Field cancerisation of the oral cavity: comparison of the spectrum of molecular alterations in cases presenting with both dysplastic and malignant lesions. Oral Oncol. 1997;33(5):332–7. 9415332 10.1016/s1368-8375(97)00035-3

[87] Kujan O, Shearston K, Farah CS. The role of hypoxia in oral cancer and potentially malignant disorders: A review. J Oral Pathol Med Off Publ Int Assoc Oral Pathol Am Acad Oral Pathol. 2017;46:246–52. 10.1111/jop.12488 27560394

[88] Tobe-Nishimoto A, Morita Y, Nishimura J, Kitahira Y, Takayama S, Kishimoto S, Matsumiya-Matsumoto Y, Matsunaga K, Imai T, Uzawa N. Tumor microenvironment dynamics in oral cancer: unveiling the role of inflammatory cytokines in a syngeneic mouse model. Clin Exp Metastasis. 2024;41(6):891–908. 39126553 10.1007/s10585-024-10306-1 PMC11607012

[89] Pigossi SC, Oliveira JA, de Medeiros MC, Soares LFF, D’Silva NJ. Demystifying the link between periodontitis and oral cancer: a systematic review integrating clinical, pre-clinical, and in vitro data. Cancer Metastasis Rev. 2025;44(3):67. 40924302 10.1007/s10555-025-10285-z PMC12420769

[90] Li R, Hou M, Yu L, Luo W, Liu R, Wang H. Association between periodontal disease and oral squamous cell carcinoma: a systematic review and meta-analysis. Br J Oral Maxillofac Surg. 2023;61(6):394–402. 37308334 10.1016/j.bjoms.2023.05.004

[91] Belibasakis GN, Seneviratne CJ, Jayasinghe RD, Vo P-D, Bostanci N, Choi Y. Bacteriome and mycobiome dysbiosis in oral mucosal dysplasia and oral cancer. Periodontol 2000. 2024;96:95–111. 38501658 10.1111/prd.12558 PMC11579824

[92] Reyes M, Urra H, Peña-Oyarzún D. Evaluating the link between periodontitis and oral squamous cell carcinoma through Wnt/β-catenin pathway: a critical review. Front Oral Health. 2025;6:1575721. 40421179 10.3389/froh.2025.1575721 PMC12104182

[93] Bacci C, Vanzo V, Frigo AC, Stellini E, Sbricoli L, Valente M. Topical tocopherol for treatment of reticular oral lichen planus: a randomized, double-blind, crossover study. Oral Dis. 2017;23(1):62–8. 10.1111/odi.12573. 27543905 10.1111/odi.12573

